# The effect of mindfulness-based intervention on depression in family caregivers of dementia patients: a meta-analysis and GRADE evaluation

**DOI:** 10.3389/fpsyg.2026.1821354

**Published:** 2026-05-12

**Authors:** Xiameng Zhang, Xiangeng Zhang, Hongyan Wang, Xiaohui Wang, Jun Meng, Yilin Yang, Chuang Zhang

**Affiliations:** 1 Department of Traditional Chinese Medicine, Affiliated Hospital of Chengdu University, Chengdu, China; 2 Sichuan Nursing Vocational College, Chengdu, China; 3 Department of Nursing, Affiliated Hospital of Chengdu University, Chengdu, China; 4 Department of Urology, Hospital of Chengdu University of Traditional Chinese Medicine, Chengdu, China

**Keywords:** caregiver, dementia, depression, family, GRADE, meta, mindfulness

## Abstract

**Objective:**

To evaluate the efficacy of MBI on depression among family caregivers of dementia patients, and to analyze the impact of intervention duration, follow-up period, and intervention methods on treatment outcomes.

**Methods:**

Search Embase, The Cochrane Library, CINAHL, PubMed, SinoMed, CNKI, Wanfang, and VIP databases for randomized controlled trials on MBI for depression in family caregivers of dementia patients. The search period spans from the inception of each journal to November 10, 2025. Meta-analysis, Egger’s test, and sensitivity analysis were performed using RevMan 5.4 and Stata 12.0 software.

**Results:**

A total of 10 RCTs involving 563 participants were included. Meta-analysis results indicated that MBI effectively improved depressive symptoms among family caregivers of dementia patients (SMD = −0.66, 95% CI (−0.83, −0.49), *Z* = 7.57, *p* < 0.00001); Follow-up analysis indicated that the intervention remained effective at 3 months post-intervention (SMD = −0.32, 95% CI (−0.62, −0.03), *Z* = 2.13, *p* < 0.05), but no therapeutic effect was observed 6 months after the intervention (SMD = −0.89, 95% CI (−1.88, 0.09), *Z* = 1.78, *p* > 0.05). Subgroup analysis revealed that 8-week MBSR, mindfulness meditation, and ACT were effective (SMD = −0.62, 95% CI (−0.85, −0.39), *Z* = 5.24, *p* < 0.00001), while 10-week MBCT did not demonstrate efficacy (SMD = −0.69, 95% CI (−1.42, 0.03), *Z* = 1.88, *p* > 0.05). GRADE assessment indicated moderate certainty of the evidence.

**Conclusion:**

MBI can improve depressive symptoms among family caregivers of dementia patients. The format of mindfulness, treatment duration, and follow-up timing influence the effectiveness of such interventions. Clinicians should prioritize selecting appropriate MBI protocols and formats, along with ensuring continuity of care and follow-up, to safeguard the sustained mental health of this vulnerable population.

**Systematic review registration:**

This systematic review was registered with PROSPERO (registration number: CRD420251208383, registration date: November 10, 2025).

## 1 Introduction

Dementia refers to a comprehensive, chronic, progressive, and irreversible decline in intellectual abilities, primarily affecting cognitive function, emotional well-being, and social behavior (Walker et al., 1998). Statistics show that approximately 46.5 million people worldwide are affected by dementia, a number projected to exceed 131.5 million by 2050 (Berk et al., 2018). Family members are the primary caregivers for dementia patients, with the caregiving period lasting up to 6.5 years (Haley, 1997). The long-term stress of caregiving makes family caregivers highly susceptible to physical and mental health issues (Gale et al., 2018), such as depression, social isolation, and a decline in quality of life (Antoniou et al., 2022). Mindfulness-based interventions (MBI) are mind–body programs centered on mindfulness meditation. They require participants to maintain single-pointed awareness of the present moment and observe things as they are without judgment (Bishop, 2002). Primary methods include Mindfulness-Based Stress Reduction (MBSR), Mindfulness-Based Cognitive Therapy (MBCT), Acceptance and Commitment Therapy (ACT), Dialectical Behavior Therapy (DBT), mindful eating, mindfulness-based self-compassion, and mindfulness-based art therapy (Kemper, 2010; Xiang and Xu, 2018).

In the field of dementia, MBI typically focus on MBSR, MBCT, mindfulness meditation, and ACT. MBSR was the first standardized group course based on mindfulness, developed by Jon Kabat-Zinn and colleagues in 1979. Initially designed for the management of chronic pain and stress-related issues, it was later gradually applied to psychiatric patients and community members. The course typically lasts 8–10 weeks, with most of the content focused on learning how to pay attention to bodily sensations through various mind–body exercises, such as sitting meditation, body scans, and yoga (Kabat-Zinn, 1982). MBCT was initially developed by John Teasdale and others, primarily for preventing symptom relapse in patients with depression (Teasdale et al., 2000). It combines MBSR with cognitive therapy to guide individuals in breaking free from the repetitive negative thought patterns that lead to depressive symptoms, and the program follows the structure of MBSR (Shahar et al., 2010). ACT was originally developed by Steve Hayes. It combines mindfulness, acceptance strategies, and behavioral therapy. Its core principle is “self-observation,” which involves cultivating the ability to observe internal phenomena without attachment, judgment, or attempts to change them, and separating oneself from painful thoughts, feelings, and sensations. Participants are encouraged to accept these phenomena while modifying maladaptive behaviors. It is primarily used in general outpatient psychotherapy for adults, and its therapeutic strategies are essentially the same as other mindfulness skills (Hayes and Wilson, 1994; Baer, 2003). Mindfulness meditation, as a practice of self-awareness training, can be traced back to the ancient Indian wisdom traditions of thousands of years ago. It is a heterogeneous set of psychological training techniques, including exercises in attention and awareness, aimed at enhancing concentration, strengthening awareness of the present moment, and familiarizing individuals with their mental processing patterns. Its methods overlap significantly with mindfulness interventions, such as focusing on the breath, body scans, and developing awareness (Levine et al., 2017; Walsh and Shapiro, 2006). MBI can be understood in various ways; they may refer to traditional meditation practices or to various MBI. All adhere to common principles, with the core concept being “paying attention to experience in the present moment, consciously, and non-judgmentally.” Key techniques include “sustained attention training combined with open awareness/decentering,” along with attitudes such as acceptance, curiosity, and patience that permeate the entire process (Gupta et al., 2025).

Numerous studies have demonstrated the effectiveness of MBI for common psychological, physiological, and social health issues across various populations (Wielgosz et al., 2019; Zhang et al., 2021). The literature on MBI for improving depressive symptoms among family caregivers of dementia patients is also growing (Gu et al., 2015). However, no study has systematically examined the impact of intervention duration, delivery methods, or follow-up timing on treatment outcomes. Therefore, this study included randomized controlled trials (RCT) examining MBI for improving depression among family caregivers of dementia patients. The aim was to explore the efficacy of MBI for depression in this population and to analyze the effects of intervention duration, delivery method, and follow-up timing.

## 2 Methods

### 2.1 Search strategy

Reported according to the Preferred Reporting Items for Systematic Reviews and Meta-Analyses (PRISMA) guidelines (Moher et al., 2010), databases searched included Embase, Cochrane Library, CINAHL, PubMed, SinoMed, CNKI, Wanfang, and VIP. The search timeframe spanned from each database’s inception to November 10, 2025. A dual search strategy combining subject headings and free-text keywords was employed. Dementia-related keywords: dementia*, alzheimer*, FAD, ATD, cognit*; Caregiver-related keywords: caregiver*, carer*, caregive*; Mindfulness intervention-related keywords: MBSR, MBCT, Mindfulness-Based Stress Reduction, Mindfulness-Based Cognitive Therapy, meditation, MBI, mindfulness, Acceptance and Commitment Therapy, ACT. Boolean operators AND and OR were used for combined searches. Additionally, relevant review articles and gray literature were identified through manual searches.

### 2.2 Inclusion and exclusion criteria

Inclusion criteria: (1) Study type: RCT published in Chinese or English; (2) Study population: family caregivers of dementia patients who exhibit depressive symptoms, aged 18 years or older; (3) Intervention: the experimental group received MBI, including MBSR, MBCT, mindfulness meditation, and ACT. The control group received general mental health education or served as a placebo control; (4) Depression Assessment: Studies must report depression-related outcomes using recognized depression assessment scales. Exclusion Criteria: (1) Duplicate publications; (2) Studies that do not explicitly report depression outcome data, or where data cannot be converted into means (M) and standard deviations (SD).

### 2.3 Grouping of MBI

The MBI included in this study is primarily divided into four categories: (1) Unstructured mindfulness meditation, which includes breathing exercises, seated meditation, relaxation training, and mindfulness yoga—these can be practiced individually or in combination. These interventions lack a unified, standardized implementation protocol. (2) Structured MBI, comprising three categories: MBSR, MBCT, and ACT. These three types of interventions all feature fixed course durations, standardized implementation frameworks, and operational procedures. Given the differences in intervention content and course structure, the studies included in this review were divided into the four independent subgroups mentioned above for analysis and publication bias assessment, in order to explore the varying effects of MBI on depression outcomes among family caregivers of dementia patients.

### 2.4 Data extraction

Data were extracted independently by two researchers according to the *Cochrane Handbook*, including: study type, publication year, author information, subject characteristics (age, gender, relationship to patient, duration of care, etc.), intervention measures, sample size, intervention duration, follow-up period, and depression data. Unreported data were obtained by contacting authors via email. In cases of disagreement, a third researcher served as the arbitrator.

### 2.5 Risk of bias assessment

Independent assessments were conducted according to the *Cochrane Handbook* (Higgins et al., 2021) quality evaluation criteria, including: random sequence generation, allocation concealment, blinding of participants and personnel, blinding of outcome assessors, completeness of outcome reporting, potential for selective reporting of results, and other biases. Each study was individually rated as low risk, high risk, or unclear risk of bias. Additionally, study results were graded using the GRADE (Brignardello-Petersen et al., 2020) system for evidence quality and recommendation strength into four levels: very low, low, moderate, and high. Assessment covered risk of bias in the literature, inconsistency, indirectness, imprecision, and risk of publication bias. Disagreements were resolved by a third researcher.

### 2.6 Statistical analysis

Meta-analyses were conducted using Cochrane Review Manager software (version 5.4), with sensitivity analyses and publication bias assessments performed using Stata software (version 12.0, developed by the American Center for Computer Resources). Data were pooled using standardized mean differences (SMD) with 95% confidence intervals. Heterogeneity was examined via chi-square tests and *I*^2^ tests, with *I*^2^ used to assess the degree of heterogeneity. A fixed-effect model was used when heterogeneity was low (*p* > 0.1, *I*^2^ < 50%); a random-effects model was selected when heterogeneity was high (*p* < 0.1, *I*^2^ ≥ 50%). Additionally, publication bias was assessed using the Egger’s test and funnel plot analysis, and sensitivity analysis was performed to evaluate the robustness of the pooled results.

## 3 Methods

### 3.1 Research screening results

**Figure 1:**
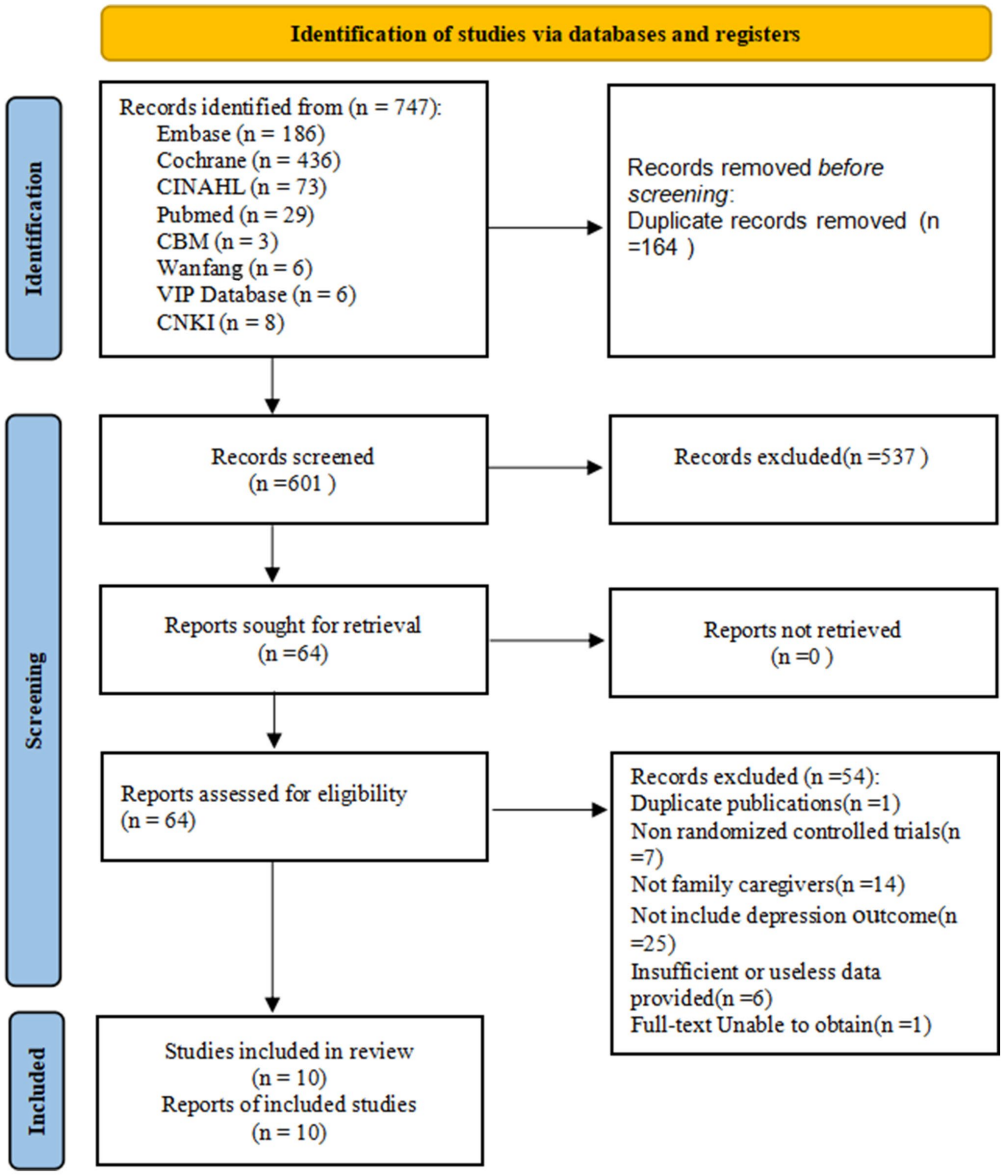
Flowchart of trial inclusion.

A preliminary search identified 747 studies. First, 164 duplicate references were excluded using EndNote X9. Subsequently, 537 studies were excluded based on title and abstract review, and an additional 54 studies were excluded after full-text review. Ultimately, 10 randomized controlled trials (Brown et al., 2016; Danucalov et al., 2013; Hou et al., 2014; Kor et al., 2019; Kor et al., 2021; Lavretsky et al., 2012; Losada et al., 2015; Oken et al., 2010; Waelde et al., 2017; Whitebird et al., 2013) were included, as shown in Figure 1.

### 3.2 Study characteristics

A total of 563 family caregivers were included, comprising 293 in the experimental group and 270 in the control group. Sample sizes ranged from 8 to 64 participants, with an average age exceeding 50 years. The intervention period lasted 8 to 10 weeks, with follow-up assessments conducted at 3 months (Brown et al., 2016; Hou et al., 2014; Kor et al., 2019) and 6 months (Kor et al., 2021; Losada et al., 2015) post-intervention. Mindfulness interventions included MBSR, MBCT, meditation, yoga, and ACT. Control groups received routine education or no intervention (blank control). Two studies (Kor et al., 2019; Kor et al., 2021) reported clinical trial registration numbers, while one study (Brown et al., 2016) did not report ethics review. Participant dropout rates during follow-up ranged from 0 to 49.46%, with Whitebird et al. (2013) not specifying reasons for withdrawal. The depression assessment scales used in the 10 studies included the POMS, BDI, CES-D, and HAMD, as shown in Table 1.

**Table 1:** Study characteristics.

Author (Year, Country)	Recruitment method	Family relationship	Duration of care (E/C)	Intervention methods (E/C)	Duration	Sample size (E/C)	Scale
Brown et al. (2016), America	Internet, Radio, Advertising, Posters, Flyers	Spouse, Sibling, Children, Grandchildren	48.22 ± 35.1 months/44.24 ± 27.6 months	MBSR/Usual education	8 weeks	Baseline (23/15), Posttreatment (19/15), 3 months follow-up (19/15)	POMS
Danucalov et al. (2013), Brazil	Radio, Newspaper	Family	4.2 ± 3.3 years/5.7 ± 3.7 years	Mindfulness meditation and yoga/No treatment	8 weeks	Baseline (25/21), Posttreatment (25/21)	BDI
Hou et al. (2014), China	Multiple strategies	Spouse, Children, Parents	Not reported	MBSR/Usual education	8 weeks	Baseline (70/71), Posttreatment (64/57), 3 months follow-up (61/52)	CES-D
Kor et al. (2019), China	Convenient sampling	Spouse, Parents, Parents-in-law Sibling	81.8 ± 77.2 months/68.4 ± 82.1 months	MBCT/Usual education	10 weeks	Baseline (18/18), Posttreatment (17/18), 3 months follow-up (16/15)	CES-D
Kor et al. (2021), China	Social media platforms (e.g., Facebook), Newsletters, Mails	Relatives by blood or marriage	68.9 ± 98.4 months/73.0 ± 85.4 months	MBCT/Usual education	10 weeks	Baseline (56/57), Posttreatment (51/53), 6 months follow-up (50/51)	CES-D
Lavretsky et al. (2012), America	Not reported	Children, Spouse	4.7 ± 2.4 years/4.2 ± 2.9 years	Mindfulness meditation/Usual education	8 weeks	Baseline (25/20), Posttreatment (23/16)	HAMD
Losada et al. (2015), Spain	Internet advertising	Family	3.3 ± 2.02 years/5.05 ± 3.06 years	ACT/Usual education	8 weeks	Baseline (45/48), Posttreatment (33/31), 6 months follow-up (25/22)	CES-D
Oken et al. (2010), Portland	Not reported	Spouse, Children	Not reported	Mindfulness meditation/Usual education	8 weeks	Baseline (10/10), Posttreatment (8/9)	CES-D
Waelde et al. (2017), America	Not reported	Family	8.23 ± 6.6 h/day	Mindfulness meditation/Usual education	8 weeks	Baseline (16/15), Posttreatment (16/15)	CES-D
Whitebird et al. (2013), America	Community outreach, Advertising, News report, Word of mouth	Children, Spouse, Sibling, Friend	51.1 ± 36.0 months/46.7 ± 42.5 months	MBSR/Usual education	8 weeks	Baseline (38/40), Posttreatment (37/35), 4 months follow-up (35/35)	CES-D

E/C, Experimental group/Control group; MBSR, Mindfulness-Based Stress Reduction; MBCT, Mindfulness-Based Cognitive Therapy; ACT, Acceptance and Commitment Therapy; POMS, Profile of Mood States; BDI, Beck Depression Inventory; CES-D, Center for Epidemiological Studies Depression Scale; HAMD, Hamilton Depression Rating Scale.

### 3.3 Risk of bias

**Figure 2:**
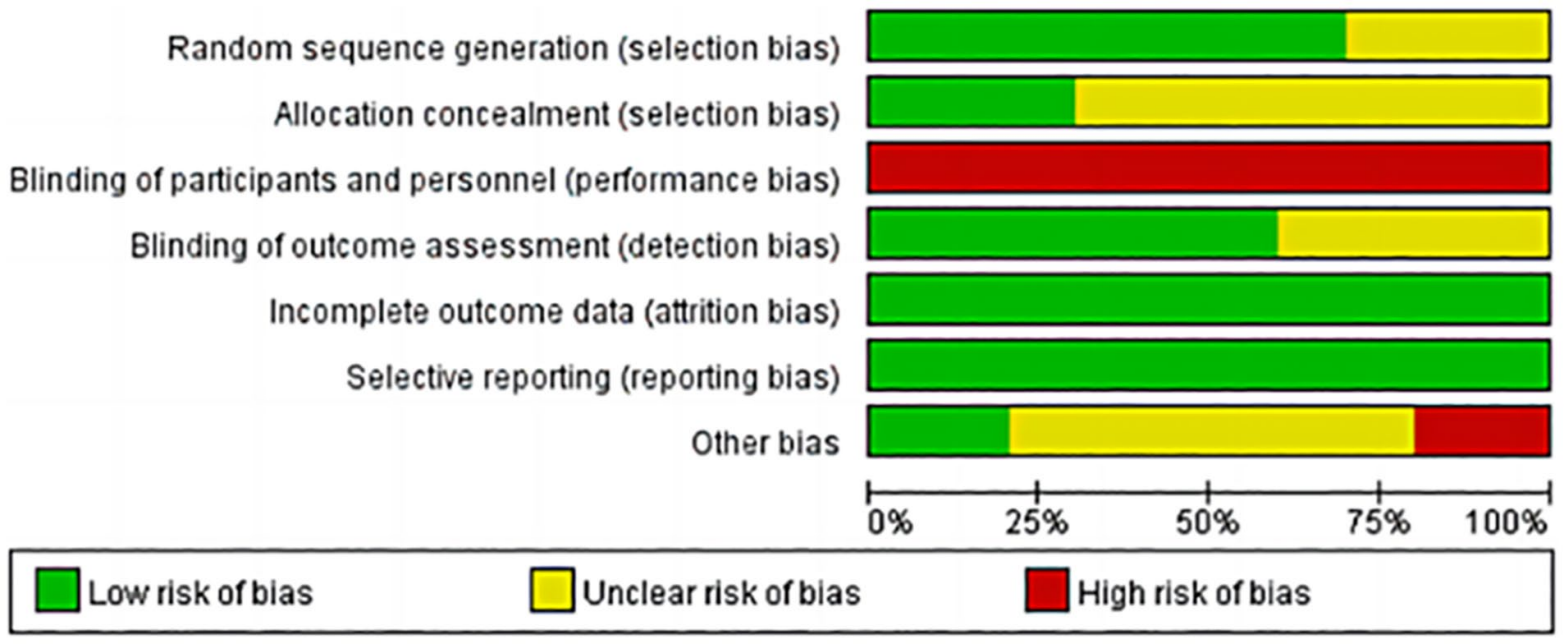
Risk of bias for included studies.

**Figure 3:**
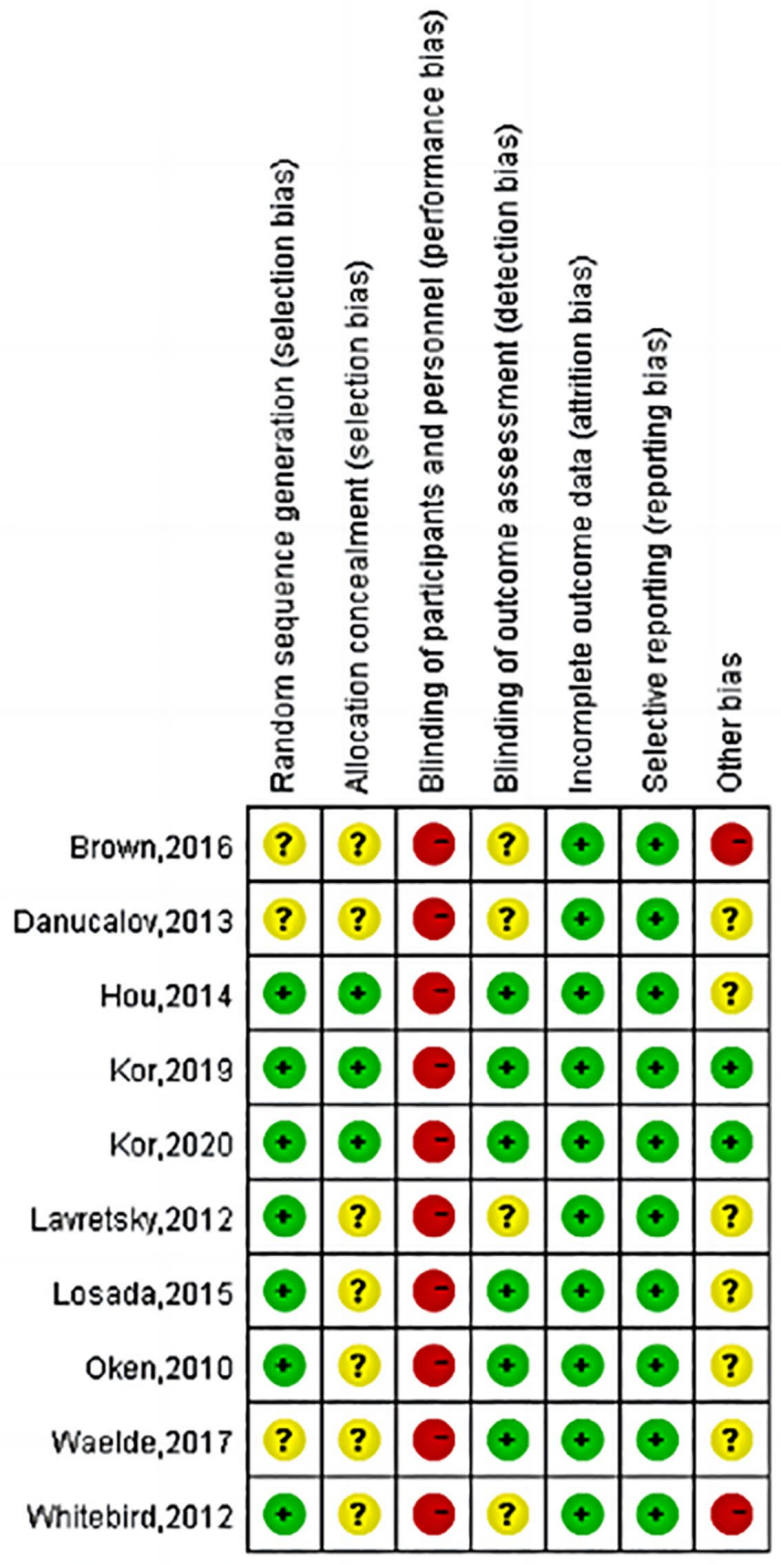
Risk of bias for included studies. Green (+) indicates a low risk of bias, yellow (?) indicates an unclear risk of bias, and red (−) indicates a high risk of bias.

Regarding random sequence generation, most studies used computer-generated random sequences (low risk), while some studies did not describe the randomization process in detail (unclear). Regarding allocation concealment, a few studies concealed the allocation scheme (low risk), while the remaining studies did not explicitly state this (unclear). Regarding blinding, due to the nature of mindfulness interventions, both participant and practitioner blinding were prone to breakdown during implementation, therefore, this was assessed as high risk (high risk). Regarding data completeness, all 10 studies reported primary outcome data (low risk). Regarding selective reporting, all 10 studies fully reported pre-specified outcome measures (low risk). Regarding other sources of bias, some studies did not disclose relevant factors such as ethics committee approval status or reasons for participant loss to follow-up, therefore, these were rated as uncertain (unclear). The remaining studies showed no apparent bias (low risk). As shown in Figures 2, 3.

According to the GRADE evidence quality grading criteria, no significant risk of bias was observed in the included studies. However, some studies did not explicitly describe blinding and randomization methods, leading to a downgrade in “risk of bias.” The total sample size exceeded 400 cases, with low overall heterogeneity (*I*^2^ = 33%). The confidence interval did not cross the clinical decision threshold (95% CI = −0.83, −0.49). All studies met the predefined inclusion criteria for PICO, resulting in an overall evidence grade of moderate certainty, as shown in Table 2.

**Table 2:** GRADE assessment.

Certainty assessment							Number of patients		Effect (95% CI)		Certainty	Importance
Number of studies	Study design	Risk of bias	Inconsistency	Indirectness	Imprecision	Other considerations	Experimental	Control	Relative	Absolute		
10	RCT	serious	Not serious	Not serious	Not serious	None	293	270	—	SMD 0.66 (−0.83 to −0.49)	⊕ ⊕ ⊕〇 MODERATE	CRITICAL

Serious: This dimension has serious flaws; Not serious: This dimension has no serious flaws; ⊕ ⊕ ⊕〇/MODERATE: The quality of evidence is moderate; there is moderate confidence in the estimated effect size, and the true effect size is likely close to the estimated effect size; CRITICAL: This outcome measure is a key outcome; it is crucial for clinical decision-making and is a core indicator influencing the recommendations.

## 4 Results of meta-analysis

### 4.1 Effect of follow-up duration on treatment outcomes

**Figure 4:**
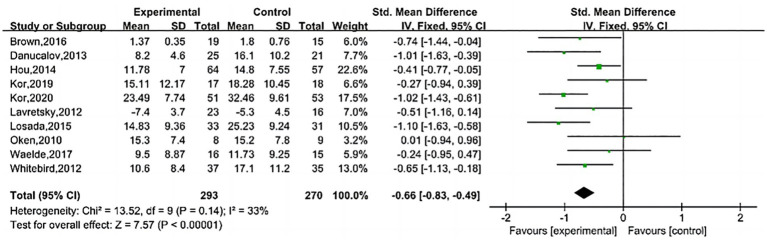
Effect of after intervention.

**Figure 5:**
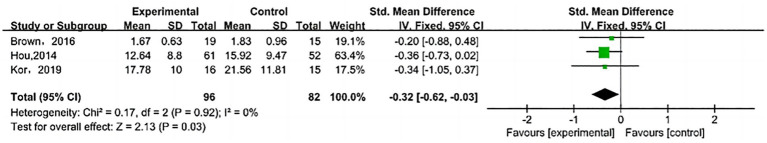
Effect of 3 months after intervention.

**Figure 6:**
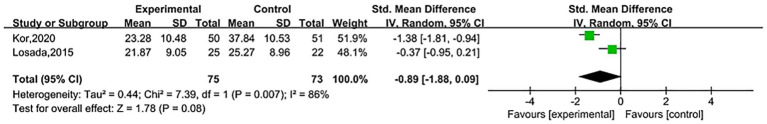
Effect of 6 months after intervention.

Meta-analysis results indicate that MBI improves depressive symptoms in family caregivers of dementia patients (SMD = −0.66, 95% CI (−0.83, −0.49), *Z* = 7.57, *p* < 0.00001). The effect was sustained at 3 months post-intervention (SMD = −0.32, 95% CI (−0.62, −0.03), *Z* = 2.13, *p* < 0.05). No effect was observed at 6 months post-intervention (SMD = −0.89, 95% CI (−1.88, 0.09), *Z* = 1.78, *p* > 0.05), as shown in Figures 4–6.

### 4.2 Effect of intervention duration on treatment outcomes

**Figure 7:**
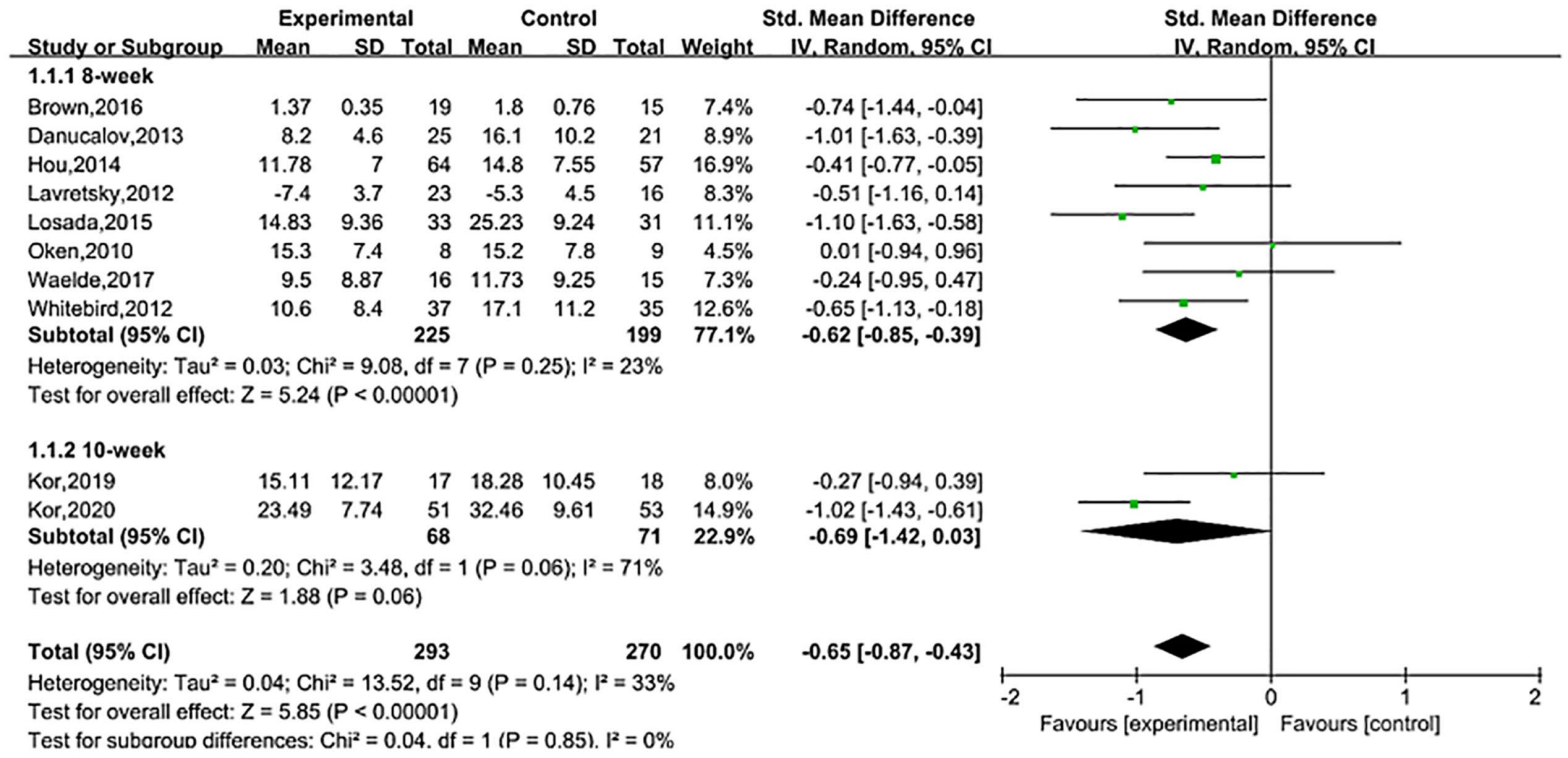
Effect of different intervention durations.

An 8-week MBI was effective (SMD = −0.62, 95% CI (−0.85, −0.39), *Z* = 5.24, *p* < 0.00001); a 10-week MBCT did not demonstrate efficacy (SMD = −0.69, 95% CI (−1.42, 0.03), *Z* = 1.88, *p* > 0.05). As shown in Figure 7.

### 4.3 Effect of intervention methods on treatment outcomes

**Figure 8:**
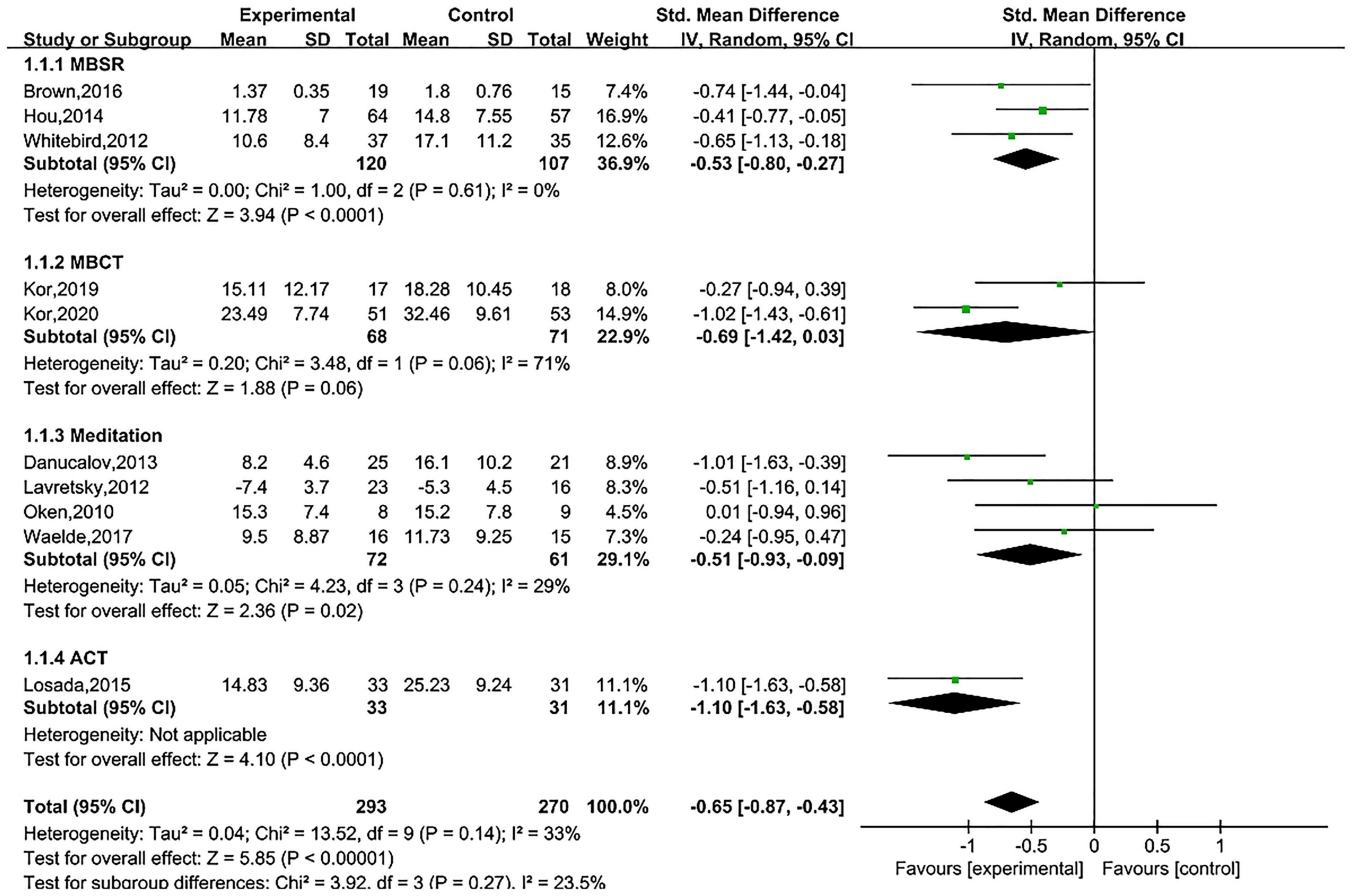
Effect of different intervention methods.

MBSR (SMD = −0.53, 95% CI (−0.80, −0.27), *Z* = 3.94, *p* < 0.0001), mindfulness meditation (SMD = −0.51, 95% CI (−0.93, −0.09), *Z* = 2.36, *p* = 0.02), ACT (SMD = −1.10, 95% CI (−1.63, −0.58), *Z* = 4.10, *p* < 0.0001) were effective for depression among family caregivers of dementia patients, while a 10-week MBCT did not demonstrate efficacy (SMD = −0.69, 95% CI (−1.42, 0.03), *Z* = 1.88, *p* = 0.06), as shown in Figure 8.

### 4.4 Publication bias

**Figure 9:**
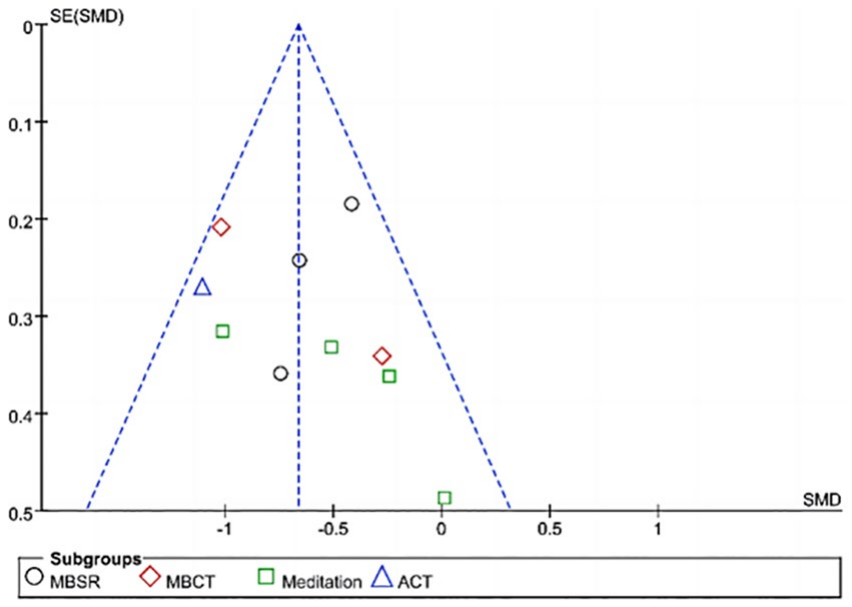
Funnel map risk of bias.

Since exactly 10 studies were included, a funnel plot was used to assess publication bias in accordance with the *Cochrane Handbook*, as shown in Figure 9. Although false-negative results are possible, an Egger’s test was also conducted to assess the risk of publication bias. The results showed *t* = 0.80 and *p* = 0.447.

## 5 Discussion

To date, the cure and symptomatic improvement of dementia remain a global challenge for the medical community. Caregivers of people with dementia face immense pressure, which affects their physical and mental health (Alzheimer's Association, 2024). As many as 80% of family caregivers of dementia patients experience symptoms of depression, and they are more prone to depression than general caregivers and the general population (Schoenmakers et al., 2010). This is attributed to factors such as the characteristics of dementia, the caregiver’s psychological state, social factors, and the nature of the relationship with the patient (Watson et al., 2019). Long-term caregiving stress and psychological issues not only increase the caregiver’s risk of developing mental health conditions but also reduce the quality of care provided to the patient (Fonareva and Oken, 2014). Therefore, alleviating the psychological distress of family caregivers caring for dementia patients is crucial to ensuring the continuity and quality of patient care (Joling et al., 2010). MBI reduces stress and anxiety in caregivers of dementia patients through emotion-focused approaches and, under certain conditions, alleviates depression. They help caregivers reduce reactivity and enhance self-regulation and acceptance when facing uncontrollable caregiving pressures and behavioral and psychological symptoms, thereby enabling them to cope with daily challenges more effectively (Kwok et al., 2025; Sapra et al., 2025).

Previous researchers have conducted relevant reviews, but conclusions remain controversial. In Liu et al. (2018) performed a systematic review on stress relief for family caregivers of dementia patients. However, their analysis included only three studies on depression (using MBSR as the intervention), and the results showed that MBSR is effective in alleviating depressive symptoms in families of people with dementia, but the quality of evidence is low (Liu et al., 2018). A study published by Ang (2020) suggested that MBSR may improve depressive and anxiety symptoms in family caregivers of dementia patients in the short term, but further research is needed to confirm these findings (Ang, 2020). This study evaluated the impact of MBI on depressive symptoms among family caregivers of dementia patients. A meta-analysis of standardized mean differences (SMD) from depression scales revealed that MBI can improve depressive symptoms in family caregivers of dementia patients (SMD = −0.66, 95% confidence interval (−0.83, −0.49), *Z* = 7.57, *p* < 0.00001, *I*^2^ = 33%). Sensitivity analysis confirmed that the pooled results remained within the 95% confidence interval after sequentially excluding studies, indicating that the results of this meta-analysis are robust and reliable. A meta-analysis of different follow-up time points showed that the efficacy of MBI persisted 3 months after the intervention [SMD = −0.32, 95% CI (−0.62, −0.03)], which is consistent with the trend of efficacy observed for MBI in depressive disorders (Li et al., 2025). However, the pooled results from three studies at 6 months post-intervention did not show an effect [SMD = −0.89, 95% CI (−1.88, 0.09)]. After ceasing mindfulness practice, regulatory and attentional abilities tend to decline, sustained practice is typically required to maintain positive effects (Shoker et al., 2024). Compared to the study by Liu et al., this study expanded the sample size and, through meta-analysis, provided evidence that MBI can improve depressive symptoms in caregivers. Additionally, the follow-up results complemented the “short-term benefit” perspective proposed by Ang et al., suggesting that the efficacy of MBI exhibits a time-dependent decline. Since the financial and caregiving burdens faced by family caregivers of dementia patients are long-term, the repeated implementation of MBI is of great significance for ensuring the sustained mental well-being of this population.

Previous meta-analyses on the mental health and caregiving burden of caregivers living with dementia patients have shown that MBI did not significantly reduce depressive symptoms overall, however, subgroup analyses suggest that MBI lasting ≥8 weeks has a positive effect on alleviating depressive symptoms in caregivers (Saragih et al., 2024). Nevertheless, this study has certain limitations. The overall pooled results exhibited high heterogeneity (*I*^2^ = 71.80%), it did not explicitly distinguish between family and non-family caregivers, and it did not conduct detailed analyses of intervention methods or follow-up periods. The subgroup analysis in this study supplements and refines the aforementioned conclusions. The results show that an 8-week MBI is effective in improving depressive symptoms among family caregivers of dementia patients (SMD = −0.62, 95% CI (−0.85, −0.39), *Z* = 5.24, *p* < 0.00001). This is consistent with the subgroup analysis results by Saragih et al., indicating that intervention duration is a key factor influencing the efficacy of MBI. In the subgroup analysis of intervention methods, the study found that MBSR, Mindfulness meditation, and ACT were all effective in improving depression. However, the two identified studies that employed MBCT (Kor et al., 2019; Kor et al., 2021) did not demonstrate efficacy (SMD = −0.69, 95% CI (−1.42, 0.03), *Z* = 1.88, *p* = 0.06). This result may be related to significant modifications to the intervention protocols, which combined the fourth and fifth sessions and adjusted the frequency of the final three sessions to once every 2 weeks. Although this was intended to reduce session time and foster daily mindfulness habits, it may have weakened immediate efficacy due to reduced intervention intensity. Additionally, the 10-week intervention period significantly increased participants’ time and financial costs, which may have inadvertently exacerbated their psychological burden, affecting adherence and the maintenance of treatment effects. However, given that this finding is based on only two studies with limited sample sizes, the robustness of these conclusions remains to be further validated by future studies with larger sample sizes and higher methodological quality.

The study used the GRADE assessment system to grade the evidence, ultimately assigning it a moderate level of certainty. This result is highly consistent with the earlier assessment of risk of bias. The primary reasons why the evidence quality did not reach a high level centered on two key issues: non-standardized allocation concealment and limitations in the implementation of blinding. These issues are closely related to the specific characteristics of studies on mindfulness interventions for family caregivers of patients with dementia and represent areas that require further exploration and improvement in future research. As a globally recognized tool for grading evidence quality, the GRADE system primarily classifies the quality of research evidence through unified and transparent standards to provide scientific support for clinical decision-making. It is widely applied across various fields to enhance the level of evidence-based nursing practice, including drug use guidelines (Zhuang et al., 2024), nursing education (Park et al., 2024), surgical procedures (Jalalzadeh et al., 2022), and chronic disease prevention and control (Zhang et al., 2022), thereby enhancing the level of evidence-based nursing practice. By standardizing evidence evaluation criteria, it drives the transition from experience-based decision-making to evidence-based practice across various medical fields. It not only provides guidance for clinical and public health practice but also encourages researchers to focus on evidence gaps and conduct higher-quality research.

## 6 Study limitations

There are minor differences among studies regarding the specific procedures, timing, and frequency of interventions, as well as the qualifications and training of teaching staff. Furthermore, due to the limited number of studies, the Egger’s test may yield false-negative results. The statistical power of subgroup analyses is also relatively low. Depression continues to be assessed using scales, although the measurement frameworks of these scales are similar and standardized mean differences (SMD) are used to account for differences in scoring ranges and the number of items, heterogeneity still exists between different scales. Furthermore, factors such as small sample sizes in some studies, a relative scarcity of follow-up studies, and potentially incomplete literature searches all undermine the ability of evidence-based research to explore true outcomes.

## 7 Conclusion

MBI effectively treats depression in family caregivers of dementia patients. The intervention method, treatment duration, and follow-up period all influence the efficacy of MBI. MBI for family caregivers of dementia patients should prioritize the selection of intervention programs and methodologies, while strengthening follow-up and ongoing treatment. The cure and symptom improvement of dementia remain a global medical challenge. Family caregivers endure immense and persistent physical and mental stress, urgently requiring continuously updated evidence-based guidelines and clinical practices to safeguard the ongoing mental health of this group.
