# Supplementary material for: The effect of mindfulness-based intervention on depression in family caregivers of dementia patients: a meta-analysis and GRADE evaluation
Source: Front Psychol. 2026 May 12;17:1821354. doi: 10.3389/fpsyg.2026.1821354 (PMC13201155; doi:10.3389/fpsyg.2026.1821354)
Supplement: Supplementary file 1 [file Data_Sheet_1.PDF]

Pubmed

Search number	Query	Sort By	Filters	Search Details	Results
4	#1 AND #2 AND #3		Abstract, Free full text, Review	(("caregiver*"[Title/Abstract] OR "carer*"[Title/Abstract] OR "caregive"[Title/Abstract]) AND ("loattrfree full text"[Filter] AND "hasabstract"[All Fields] AND "review"[Publication Type]) AND (("MBSR"[Title/Abstract] OR "MBCT"[Title/Abstract] OR "mindfulness based stress reduction"[Title/Abstract] OR "mindfulness based cognitive therapy"[Title/Abstract] OR "meditation"[Title/Abstract] OR "mindfulness"[Title/Abstract] OR ("acceptance"[Title/Abstract] AND "commitment therapy"[Title/Abstract]) OR "ACT"[Title/Abstract]) AND ("loattrfree full text"[Filter] AND "hasabstract"[All Fields] AND "review"[Publication Type])) AND (("dementia*"[Title/Abstract] OR "alzheimer"[Title/Abstract] OR "cognit*"[Title/Abstract] OR "FAD"[Title/Abstract] OR "ATD"[Title/Abstract]) AND ("loattrfree full text"[Filter] AND "hasabstract"[All Fields] AND "review"[Publication Type]))) AND ((ffrft[Filter]) AND (fha[Filter]) AND (review[Filter]))	29
3	((((dementia*[Title/Abstract]) OR (alzheimer[Title/Abstract])) OR (cognit*[Title/Abstract		Abstract, Free full text, Review	("dementia*"[Title/Abstract] OR "alzheimer"[Title/Abstract] OR "cognit*"[Title/Abstract] OR "FAD"[Title/Abstract] OR "ATD"[Title/Abstract]) AND ((ffrft[Filter]) AND (fha[Filter]) AND (review[Filter]))	44,150

	])) OR (FAD[Title/Abstract])) OR (ATD[Title/Abstract])				
2	(((((((MBSR[Title/Abstract]) OR (MBCT[Title/Abstract])) OR (mindfulness-based stress reduction[Title/Abstract])) OR (mindfulness-based cognitive therapy[Title/Abstract])) OR (meditation[Title/Abstract])) OR (mindfulness[Title/Abstract])) OR (acceptance[Title/Abstract] AND commitment therapy[Title/Abstract])) OR (ACT[Title/Abstract])		Abstract, Free full text, Review	("MBSR"[Title/Abstract] OR "MBCT"[Title/Abstract] OR "mindfulness based stress reduction"[Title/Abstract] OR "mindfulness based cognitive therapy"[Title/Abstract] OR "meditation"[Title/Abstract] OR "mindfulness"[Title/Abstract] OR ("acceptance"[Title/Abstract] AND "commitment therapy"[Title/Abstract]) OR "ACT"[Title/Abstract]) AND ((ffrft[Filter]) AND (fha[Filter]) AND (review[Filter]))	20,813
1	((caregiver*[Title/Abstract]) OR		Abstract, Free full text,	("caregiver*"[Title/Abstract] OR "carer*"[Title/Abstract] OR "caregive"[Title/Abstract]) AND	5,211

	(carer*[Title/Abstract])) OR (caregive[Title/Abstract])		Review	((ffrft[Filter]) AND (fha[Filter]) AND (review[Filter]))	

Embase

#18. #5 AND #11 AND #14 AND #17	186
#17. #15 OR #16	853,732
#16. (randomized:ti,ab,kw OR randomised:ti,ab,kw) AND controlled:ti,ab,kw AND trial:ti,ab,kw	318,778
#15. 'randomized controlled trial'/exp	757,904
#14. #12 OR #13	171,241
#13. 'caregiver*':ti,ab,kw OR 'carer*':ti,ab,kw OR 'caregive':ti,ab,kw	150,411
#12. 'caregiver'/exp	108,032

#11.	#6 OR #7 OR #8 OR #9 OR #10	419,474
#10.	'mbsr':ti,ab,kw OR 'mbct':ti,ab,kw OR 'mindfulness-based stress reduction':ti,ab,kw OR 'mindfulness-based cognitive therapy':ti,ab,kw OR 'meditation':ti,ab,kw OR 'mindfulness':ti,ab,kw OR 'acceptance and commitment therapy':ti,ab,kw OR 'act':ti,ab,kw	413,443
#9.	'acceptance and commitment therapy'/exp	2,400
#8.	'mindfulness'/exp	14,519
#7.	'meditation'/exp	10,471
#6.	'mindfulness-based stress reduction'/exp	668
#5.	#1 OR #2 OR #3 OR #4	3,578,839
#4.	'alzheimer disease'/exp	239,644
#3.	'dementia*':ti,ab,kw OR 'alzheimer*':ti,ab,kw OR 'cognit*':ti,ab,kw OR 'fad*':ti,ab,kw OR 'atd':ti,ab,kw	985,174
#2.	'cognition'/exp	2,959,625
#1.	'dementia'/exp	433,359

Cochrane

#1 (dementia):ti,ab,kw OR (alzheimer):ti,ab,kw OR (cognitIon):ti,ab,kw OR (FAD):ti,ab,kw OR (ATD):ti,ab,kw (Word variations have been searched) 108528

#2 MeSH descriptor: [Dementia] explode all trees 7943

#3 MeSH descriptor: [Cognition] explode all trees 13615

#4 (MBSR):ti,ab,kw OR (MBCT):ti,ab,kw OR (mindfulness-based stress reduction):ti,ab,kw OR (mindfulness-based cognitive therapy):ti,ab,kw OR (meditation):ti,ab,kw (Word variations have been searched) 6222

#5 (mindfulness):ti,ab,kw OR (acceptance and commitment therapy):ti,ab,kw OR (ACT):ti,ab,kw (Word variations have been searched) 51378

#6 MeSH descriptor: [Meditation] explode all trees908

#7 MeSH descriptor: [Mindfulness] explode all trees 1698
#8 MeSH descriptor: [Acceptance and Commitment Therapy] explode all trees 360
#9 (caregiver):ti,ab,kw OR (caregivers):ti,ab,kw OR (carer):ti,ab,kw OR (carers):ti,ab,kw OR (caregive):ti,ab,kw (Word variations have been searched) 20445
#10 MeSH descriptor: [Caregivers] explode all trees 3171
#11 #1 OR #2 OR #3 111402
#12 #4 OR #5 OR #6 OR #7 OR #8 53208
#13 #9 OR #10 20445
#14 #11 AND #12 AND #13 438

CINAHL

S1	SU dementia OR SU Alzheimer's Disease OR SU Cognition Disorders	21,253
S2	AB dementia* OR AB alzheimer* OR AB cognit* OR AB FAD OR AB ATD	44,014
S3	((AB dementia* OR AB alzheimer* OR AB cognit* OR AB FAD OR AB ATD) OR (S1)) AND (S1 OR S2)	53,666
S4	SU meditation OR SU mindfulness OR SU (acceptance and commitment therapy)	3,766
S5	AB MBSR OR AB MBCT OR AB mindfulness-based	21,418

	stress reduction OR AB mindfulness-based cognitive therapy OR AB meditation OR AB mindfulness OR AB (acceptance and commitment therapy) OR AB ACT	
S6	(AB S4 OR S5) AND (S4 OR S5)	23,303
S7	AB caregiver* OR AB carer* OR AB caregive	18,550

CBM

#1	"痴呆"[加权:扩展] OR "阿尔茨海默病"[加权:扩展] OR "认知功能障碍"[加权:扩展]	加权扩展检索	50227
#2	"正念"[加权:扩展] OR "冥想"[摘要:智能] OR "接纳与承诺疗法"[摘要:智能]	加权扩展 + 智能检索	11034
#3	"照顾"[摘要:智能] OR "护理人员"[摘要:智能]	智能检索	93579
#4	(((#1) AND (#2)) AND (#3))	组合检索：痴呆 / 认知障碍 AND 正念 / 冥想 / ACT AND 照顾 / 护理人员	3

Wangfang

1	摘要：(照顾者 or 护理人员)	摘要	157136
2	摘要：(痴呆) or 摘要：(阿尔兹海默病) or 摘要：(阿尔莫兹海默病) or 摘要：(认知功能障碍)	摘要	73846
3	摘要：(正念) or 摘要：(冥想) or 摘要：(接纳与承诺疗法)	摘要	7998
4	摘要：(照顾者 or 护理人员) and 摘要：(痴呆 or 阿尔兹海默病 or 阿尔莫兹海默病 or 认知功能障碍) and 摘要：(正念 or 冥想 or 接纳与承诺疗法)	摘要（三字段 AND 组合）	6

VIP

1#	((((摘要=正念 OR 摘要=冥想) OR 摘要=接纳与承诺疗法) AND ((摘要=痴呆 OR 摘要=阿尔兹海默病) OR 摘要=阿尔茨海默病 or 认知功能障碍)) AND (摘要=照顾者 OR 摘要=护理人员))	摘要字段	6
2#	(((((题名或关键词=照顾者 OR 题名或关键词=护理人员) AND ((题名或关键词=正念 OR 题名或关键词=冥想) OR 题名或关键词=接纳与承诺疗法))) AND (((题名或关键词=痴呆 OR 题名或关键词=阿尔兹海默病) OR 题名或关键词=阿尔莫兹海默病) OR 题名或关键词=认知功能障碍)))	题名或关键词字段	6

3#	((((题名或关键词=照顾者 OR 题名或关键词=护理人员) AND ((题名或关键词=正念 OR 题名或关键词=冥想) OR 题名或关键词=接纳与承诺疗法)) AND (((题名或关键词=痴呆 OR 题名或关键词=阿尔兹海默病) OR 题名或关键词=阿尔莫兹海默病) OR 题名或关键词=认知功能障碍))	题名或关键词字段	6

CNKI

((((((旧版主题='正念') OR (旧版主题='冥想')) OR (旧版主题='接纳与承诺')) AND (((旧版主题='正念') OR (旧版主题='冥想')) OR (旧版主题='接纳与承诺'))) AND ((((旧版主题='痴呆') OR (旧版主题='阿尔兹海默病')) OR (旧版主题='阿尔莫兹海默病')) OR (旧版主题='认知功能障碍'))) AND ((旧版主题='照顾者') OR (旧版主题='护理人员')))
